# NEWS - REGIONS

**DOI:** 10.7189/jogh.05.020201

**Published:** 2015-12

## AFRICA

➢ The European Medicines Agency has cleared the world’s first malaria vaccine, prior to its approval for use in Africa. The vaccine (Mosquirix) was developed by GlaxoSmithKline, who have pledged not to profit from the vaccine. It is designed to target infection in children in Africa, although it does not appear to be very effective in protecting young babies – children aged 5–17 months seem to benefit the most, with a 33% reduction in cases over four years. Unfortunately, the vaccine cannot be administered alongside other childhood vaccines, and children must receive all shots (including a booster shot) to benefit. However, even a partially effective vaccine can help countries with high infection rates. Prof Adrian Hill of the Jenner Institute, Oxford said “a bed net is more effective that this vaccine, but nonetheless it is a very significant scientific achievement. I see it as a building block towards much more effective malaria vaccines in years to come.” (*BBC*, 24 Jul 2015)

➢ The Islamic Development Bank (IBD) has pledged US$ 360 million of financing to support the post–Ebola recovery effort in Sierra Leone and Guinea. The announcement was made at the UN–organised International Ebola Recovery Conference, held in New York in July 2015. Guinea would receive US$ 220 million and Sierra Leone US$140 million. Liberia is not a member country of the IBD, but the IDB will support post–Ebola recovery by strengthening partnership through other agencies such as the Arab Co–operation Group. The late King Abdullah of Saudi Arabia had also donated US$ 35million to the IBD to support the countries affected by Ebola. (*allafrica.com*, 28 Jul 2015)

➢ The global disease burden for conditions treatable by surgery is twice that of HIV/AIDS, malaria and TB combined – and 1–in–3 of the world’s population has no access to surgery. Surgery is also dangerous without appropriate equipment and training–including basic equipment like pulse oximeters, which risks 6 million lives each year. Uganda is working to rectify this by introducing several innovations and improvements. First, surgeons are using simple, cheap and innovative drill covers to perform orthopaedic surgery using household drills. The NGO Lifebox has distributed cheap and simple pulse oximeters across Ugandan hospitals. Ugandan hospitals are also adopting the WHO’s surgical safety checklist, which can cut death rates and surgical complications by more than 30% by reducing the most common errors. (*Al Jazeera*, 17 Aug 2015)

➢ South–east Sierra Leone has been free of Ebola for six months, whereas it continues to linger in the north–west of the country. This is puzzling, as the north–west has received more foreign aid. The south–east is more isolated, which may explain part of its success, but other factors could be more important. In the south–east, prominent locals, doctors, priests, political leaders and chiefs played larger roles in fighting Ebola. In the north–west, Ebola efforts were managed by a politically–appointed co–ordinator, who side–lined non–medical people and traditional healers. By summer 2015 this had improved, with agencies working more closely with traditional and political rulers. Chief Vangahun, one of the south–east’s paramount chiefs at the forefront of the region’s battle against Ebola, commented that “pouring money into the fight against Ebola does not solve the issue.” (*The Economist*, 29 Aug 2015)

➢ The WHO’s global status report on road safety shows that 1.25 million people die each year from road traffic accidents. The risks of dying largely depends on where people live and how they move around. Africa has the highest death rate from road accidents, although all low–and middle–income countries are disproportionately affected by road deaths, despite having fewer vehicles per capita. Countries with the most success in reducing this toll have introduced better legislation, stricter enforcement, and safer roads and vehicles. Although road deaths are stabilising, more must be done to tackle these generally–preventable deaths, including improved public transport and preventing pedestrian deaths and injuries by making cycling and walking safer. “Road traffic fatalities take an unacceptable toll–particularly on poor people in poor countries,” says Dr Margaret Chan, the WHO Director–General. (*Ghana Business News*, 19 Oct 2015)

## ASIA

➢ According to the Internal Displacement Monitoring Centre’s (IDMC) annual report, 19 million people were displaced by natural disasters in 2014 (eg, floods and earthquakes), a decrease from 32 million in 2012. More than 90% of these displacements were in developing countries, with China, India and the Philippines experiencing the most displacement. This disguises a rising trend, as the average number of people displaced each year for every million inhabitants has doubled since 1970. The IDMC statistics may under–report smaller incidents, and slower–onset incidents (eg, environmental degradation and drought) are not included. The IDMC states that improved monitoring and data are essential to measure the effectiveness of disaster management initiatives and development, especially due to the growing risks posed by urbanisation and climate change. However, the rise in displacement may mask a more positive trend – fewer people are dying in natural disasters, with the Philippines in particular improving in pre–emptive evacuation. (*scidev.net*, 30 Jul 2015)

➢ Following a meeting with government officials, opposition party leaders and ethnic minority groups, Myanmar announced an ambitious target to eliminate malaria in the country by 2030 – at an estimated cost of US$ 1.2 billion. The range of stakeholders reflects a rare consensus over a major challenge. Malaria is a huge burden on Myanmar’s people and economy. Myanmar is a bridge between south–east Asia–where strains resistant to artemisinin first took hold–and India, from which it could spread to Africa and beyond. Malaria cases have fallen from 300 000 in 2013 to 250 000 in 2014, as Myanmar moves from control to eradication. Ahead of the country’s elections in Nov 2015, the leader of the National League of Democracy, the main opposition party affirmed his support. “Whoever wins the coming election, this malaria elimination program will go on, because there will still be this disease threatening our country,” said Dr Tin Myo Win. (*The Irrawaddy*, 5 Aug 2015)

➢ According to UNICEF’s *Promise Renewed: 2015 Progress Report*, Indonesia has made substantial progress in reducing child mortality. In 1990, there were 85 deaths per 1000 births for children aged under 5 years, falling to 27 per 1000 births by 2015. This represents 5 million lives saved over the period. This success is due to expanding immunisation, promoting breast–feeding, the prompt diagnosis and treatment of common childhood illnesses–all underpinned by strong economic growth. “Saving the lives of millions of children is one of Indonesia’s great achievements over the past 25 years,” said UNICEF representative Gunilla Olsson. She noted that further reductions will depend on addressing other issues, such as premature birth, severe infections and asphyxia. (*TEMPO*, 9 Sept 2015)

➢ Médecins sans Frontières (MSF) is calling for an independent humanitarian commission to investigate the bombing of an MSF hospital in Kunduz, Afghanistan, regarding it as a war crime. The bombing killed 22 people, and left thousands of people without health care. The US military took responsibility for the air strike, admitting that it was a mistake. President Obama apologised directly to the MSF president Dr Joanne Liu, and offered his condolences. This would be the first time that such a commission (created under the Geneva Convention in 1991) will have been activated. The USA has launched a separate investigation into the bombings. However, “if we let this go, as if it was a non–event, we are basically giving a blank cheque to any countries who are at war. If we don’t safeguard that medical space for us to do our activities, then it is impossible to work in other contexts like Syria, South Sudan, like Yemen,” says Dr Liu. (*Reuters*, 7 Oct 2015)

➢ North Korea received US$ 21.3 million in humanitarian aid in the first six months of 2015 (US$ 9.64 million of food aid, US$ 6.2 million of health care, and US$ 2.4 million of drinking water). However, there are severe shortages of medicine in the country, with more than 80% of village clinics suffering from chronic shortages of supplies, despite the state’s guarantee of free universal health care. This has led to counterfeit versions of donated medicines flooding local markets. These fake medicines, labelled as “UN medicines” to disguise their origins, are dangerous to patients, and undermine trust in genuine donated supplies. Much donated aid has also been seized by the country’s elite, leading to debate about the wisdom of sending aid. However, others argue that some supplies will eventually reach the wider population, thus providing some benefit. (*Radio Free Asia*, 2 Nov 2015)

## AUSTRALIA AND WESTERN PACIFIC

Experts warn that Australia is experiencing an epidemic of hepatitis C, with nearly 250 000 people infected by the blood–borne virus. 90% of new infections are amongst injecting drug–users, despite increased efforts to ensure access to clean syringes. Hepatitis C can remain undetected for years, but if left untreated it will attack the liver and can lead to cirrhosis, liver disease and liver cancer. Although treatment options are improving, they are expensive and many are afraid of the side effects. An estimated 10 000 people contract the virus each year, and 700 people die from the associated liver diseases. Drugs support groups call for improved access to clean injecting equipment, such as more access points, after–hours access, increased education and removing legal barriers to the peer distribution of injecting equipment. (*ABC*, 28 Jul 2015)

➢ The Trans–Pacific free trade agreement currently being negotiated by 12 Pacific Rim countries (Australia, Brunei, Canada, Japan, Malaysia, Mexico, New Zealand, Peru, Singapore, the US and Vietnam) could threaten the affordability of generic medicines, and undermine the HIV response in developing countries. The Foundation for AIDS Research (amfAR) warned that the TPP could delay generic drugs becoming available on the market by expanding intellectual property protection for existing pharmaceutical drugs, hence keeping prices high. Generic medicines have been crucial in expanding the antiretroviral treatment for HIV–positive people in developing countries. “If the TPP moves forward, it will set a dangerous global precedent and put life–saving drugs beyond the reach of millions of people with HIV/AIDS, cancer, tuberculosis and hepatitis C,” said Kevin Robert Frost of amfAR. (*The People’s World*, 25 Aug 2015)

➢ Australia is the only developed country not to have eliminated trachoma, an infectious eye disease. Trachoma is endemic amongst Australia’s remote Indigenous communities. It causes inverted eye–lashes which painfully scratch the cornea and could eventually cause blindness. Nick Martin, the Director of Public Affairs at the Fred Hollows Foundation, believes that trachoma is part of a bigger issue for Indigenuous people, and that gaps in housing and access to services for Indigenous people must be closed. Globally, the WHO is aiming to eliminate trachoma by 2020. “It remains a disgrace that trachoma is still endemic in Indigenous communities in Australia; it should not be happening in the 21st century and that is why we have to press onto 2020 for elimination,” says Nick Martin. (*The Diplomat*, 3 Sept 2015)

➢ Australia’s highest court has ruled that BRCA–1, a breast–cancer gene, cannot be patented. This follows an appeal by Yvonne D’Arcy, an Australian breast cancer survivor, against a US–biotechnology company, Myriad Genetics. Ms D’Arcy argued that allowing corporations to own human gene patents stifled cancer research, the development of treatments for genetic diseases, and that Myriad could charge high prices for testing for the BRCA1 mutation. Myriad’s counter–argument is that patents ensure innovation could be commercialised for everyone’s benefit. Prior to this case, the US Supreme Court had ruled that genes are not patentable. (*Xinhua*, 7 Oct 2015)

➢ At a summit in Fiji ahead of UN climate talks in December, the Pacific Island nations called for the world to address the health impacts of climate change on these islands. The Fijian health minister, Rata Inoke Kubuabola, said that Fiji was dealing with re–emerging climate–influenced diseases eg, typhoid, dengue fever, leptospirosis etc. In 2014, dengue fever infected 20 000 people in Fiji. Almost all Pacific Island nations are expected to attend the meeting, where they will call for the world to act decisively to address climate change. They are critical of industrialised nations who they perceive to risk the entire planet to protect their economies and standard of living, at the expense of the Pacific Islands and other parts of the world–who are not responsible for climate change. “We say if you save Tuvalu you save the world, because if you bring down emissions enough to save us, the rest of the world will be OK,” says Santini Tulaga Manuella, Tuvalu’s health minister. (*The Guardian*, 2 Nov 2015)

## CHINA

➢ Universal health coverage is unusual in developing countries, but China went against this trend by implementing universal health insurance coverage (UHIC) in 2011. In a paper published in *Health Policy,* Hao Yu argues that this coverage, the largest–ever expansion of UHIC, was driven by several factors. First, the SARS outbreak in 2003 highlighted the country’s under–investment in health. There is strong public support for government intervention in health care, coupled with increased political support for addressing health care. UHIC is heavily subsidised, with the government covering 75–85% of costs; and China’s strongly–performing economy enables this investment in UHIC. Local governments were given targets and incentives to extend coverage and enrolment, including their own performance evaluation. Lastly, China used a successful two–pronged approach to implementing UHIC, first achieving wide but shallow coverage, then expanding benefits. China now faces the challenges common to UHIC systems–quality improvement and cost control. (*Health Policy*, 28 Jul 2015)

➢ China’s government has launched a new three–year programme to cut the number of under–age drug users, and to raise awareness amongst young people. The average age of drug users in China has fallen, and 1.89 million registered drug users are younger than 35 years–58% of the total number of registered users. The programme will target young people aged 10–25 years, and its large–scale awareness campaign will reach all students. It aims to keep the number of underage drug offenders at less than 0.3% of all drug offenders, and achieve a “notable reduction” in new users. Liu Yuijin, the deputy director of China’s National Narcotics Control Commission, pledged that the government will treat the programme as a priority over the next three years. (*Xinhau*, 30 Jul 2015)

➢ According to a report published by the consultancy firm Clearstate, the health care market in China is undergoing structural change driven by health care reforms, market dynamics and the changing needs of patients in China. Clearstate predicts five key trends that will affect the health care sector by 2020. First, new service providers will emerge, driven by private insurance creating demand for services beyond public hospitals. Second, China’s private insurance market is set to grow by 25–30% for the next 5 years, fuelled by an increasing number of wealthier individuals and enterprises. Third, the government classifies the medical device industry as a priority industry, and plans to support strong domestic pharmaceutical companies. Fourthly, there will be increased emphasis on prevention (eg, screening programmes for chronic diseases) and health management. Lastly, China is set to become the world’s second–largest mobile–health market, growing by 60% from 2015 to 2018. This is driven by people’s need for reliable health information beyond the limited amount doctors can provide physicians being unable to provide time beyond diagnosis and treatment, and the use of mobile health in rural areas to overcome accessibility difficulties. (*Economist Intelligence Unit*, 5 Oct 2015)

➢ Taiwan’s Centers for Disease Control reported suspected 19 deaths from dengue fever. Of the 19 suspected deaths, 18 were from Tainan and one from Kaohsiung. 18 of the 19 people also suffered from chronic diseases. Dengue fever has been especially prevalent in Tainan in 2015, with 3234 confirmed cases this year, accounting for most of the 3686 cases reported throughout Taiwan since 1 May 2015. The government is spreading pesticides and pumping seawater into drainage systems to control the mosquito population which spreads the disease. (*China Post*, 2 Sept 2015)

➢ Parts of China was covered in smog as levels of the most dangerous particulate PM2.5 reached the highest recorded levels for 2015, and were almost 50 times higher than the WHO’s recommended limits. 21 cities experienced high levels, with the Shenyang city government issuing a “level 1 high alert” emergency response, which includes schools banning outdoor activities, asking residents to use “green transport”, and to stay indoors and take health precautions. China’s air pollution is linked to hundreds of thousands of early deaths, and can cause heart disease, stroke and lung diseases. The smog was caused by a surge in coal–powered heating ahead of winter, and heavy pollution being blown in from other provinces. (*South China Morning Post*, 9 Nov 2015)

## EUROPE

➢ European governments are working together to negotiate prices for pharmaceuticals, in the hope that the higher volumes will lead to lower prices. Current partnerships are responding to particular domestic circumstances, but the formation of these bargaining blocs has led to renewed calls for an EU–wide way of negotiating with pharmaceutical companies to help reduce high drug prices. Industry bodies believe that such negotiations should be national, although they admit there is scope for some flexibility. There are advantages in an EU–wide pricing system, but the chances of reaching agreeing on one are remote because health care is seen as a national issue, and the difference between EU economies is too great. From a pharmaceutical perspective, a single European decision–making process would increase the risks of drug development. However, the trend towards joint negotiation could spread, although pharmaceutical companies will try to separate them. (*Economist Intelligence Unit*, 4 Jun 2015)

➢ Greece’s financial crisis has had a massive impact on its health care system. State hospitals have cut budgets by up to 50%, basic items such as gloves and syringes are in short supply, and the number of doctors and nurses is critically low. Rising unemployment and poverty has left 2.5 million Greek people without health coverage, and screening programmes have been slashed, leading to more diseases being diagnosed at a later stage. Pharmacist are dealing with delays in government payments, difficulties in sourcing imported drugs due to disruptions in the supply chain, and a shortage of funds. Many Greek nationals are relying on voluntary health care staff – previously the preserve of refugees or other nationals with no health care access. “We’re already facing a humanitarian crisis in Greece. Of all the damage done during the last five years, health care has been hit the worst,” says Dr Sofia Garane, a clinic manager from the NGO Doctors of the World. (*The Guardian*, 9 Jul 2015)

➢ Research on e–cigarettes, commissioned by Public Health England and led by Prof Ann McNeill of King’s College London and Prof Peter Hajek of Queen Mary University of London, has found that they are 95% less dangerous than traditional cigarettes. E–cigarettes may also contribute to declining rates of smoking, and could be an effective intervention to reduce smoking in groups where it is highest. “While smoking cessation services continue to be the most successful way to help people stop smoking, the highest successful quit rates are being seen among smokers who are also using e–cigarettes. Providing health care professionals with accurate advice and information on their use is necessary if we are to unlock the full potential of e–cigarettes in helping people to kick their habit,” says Prof Penny Woods of the British Lung Foundation. (*Medical News Today*, 19 Aug 2015)

➢ Human rights campaigners warn that Armenia’s mental health laws are open to abuse, contain incentives to detain people unnecessarily, and make it too easy to declare people mentally incompetent. The laws are a relic from Armenia’s Soviet period, where detention in mental institutions could be used to silence trouble–makers. One phone call to the police or a psychiatric unit is sufficient to have someone hospitalised; and if the person refuses to be admitted the courts can be applied to for mandatory admission, without the patient being represented. There is no requirement to periodically review initial assessments, and high payments for hospitalisations create incentives for hospitals to admit and keep patients. Tatevik Khachatrian, Armenia’s state deputy ombudsman, calls for complete revision of the laws, saying that “we have registered cases when people with no mental illness were locked away in institutions where they were tied up and abused.” Julietta Amarikian was nearly hospitalised when her brother attempted to have her detained in a psychiatric unit following a family dispute. With the help of a human rights activist, she successfully appealed, but her case highlights the dangers of government inaction on Armenia’s arcane mental health laws. (*The Guardian*, 12 Oct 2015)

➢ According to the OECD’s annual *Health at a Glance 2015* report, the UK has one of the worst health care systems in the developed world. Too many lives are being lost because the quality of care is not improving quickly enough. Cancer survival rates are improving, but still remain in the lower third of OECD countries for some cancer types, and acute care is average. The UK had insufficient staff to ensure basic procedures are being followed, with a poor record on hospital–acquired infections. The UK also lags behind in life expectancy at birth, and in containing obesity in adults and children. This is partly due to the UK spending less on health care than other OECD countries, and too much attention on institutional structures and too little on making the basic processes of care work better. A Department of Health spokesperson claims to be prioritising investment in frontline NHS services, with an additional 10 500 doctors and 7600 nurses since May 2010, and aims to make the NHS the world’s safest health care system. *(International Business Times*, 5 Nov 2015)

## INDIA

➢ India has more malnourished people than any other country, and 30% of its children are underweight–albeit an improvement on 43% in 2002 – compared to 3% in China. Malnutrition weakens people and renders them more vulnerable to disease, and stunts brain development in children. The Indian government is making limited progress in fighting hunger, due to taboos, corruption and political pride. Measures such as free school lunches have helped, but there are problems of rotten food, stolen subsidies, and banning certain foodstuffs (eg, high–protein eggs) due to dietary restrictions. The Indian government has not published the results of a joint UN survey on child nutrition, hindering states’ ability to learn from each other. Critics suspect its suppression may be due to its criticism of Gujarat, the home state of Narendra Modi, India’s Prime Minister. The government should publish all data that could lead to better policies to tackle malnutrition, and focus on girls and women, who are more likely to be malnourished than men and boys. (*The Economist*, 4 Jul 2015)

➢ According to the WHO, the malaria parasite *P. vivax* is causing a high disease burden in India. In 2013, there was an estimated 15.8 million symptomatic cases of *P. vivax* worldwide, with 67% occurring in south–east Asia, which includes India, where over 50% of cases are caused by this parasite. However, there is a small fall in malaria cases caused by the more dangerous *P. falciparum* parasite. To date, anti–malaria efforts have focused on the *P. falciparum* strain, but it now recognised that this must be broadened to *P. vivax,* which the WHO estimates could be responsible for up to 15% of malaria deaths outside Africa. *P. vivax* is unresponsive to existing control measures, and can remain hidden and beyond the reach of current diagnostic tools and medication. “We need targeted strategies for *P. vivax* malaria which presents distinct challenges for control and elimination compared to *P. falciparum*,” says the WHO Regional Director, Poonam Khetrapal Singh. (*The Times of India*, 30 Jul 2015)

➢ There are concerns that India’s proposed changes to its intellectual property legislation could threaten the provision of cheap, life–saving drugs to sub–Saharan Africa. India protects drug–making processes not products, allowing pharmacists to “reverse engineer” drugs with different processes and offer cheaper, generic copies. However, India is being pressurised by the USA to protect data produced when developing a drug. This would outlaw compulsory licensing of (and may even restrict) generic versions of off–patent drugs. An estimated 80% of antiretroviral drugs in Africa are from India, which supplies 17.7% of Africa’s pharmaceutical imports. This helped India’s pharmaceutical industry to grow to US$ 19.36 million each year. It is argued that India should resist US pressure by reminding it that its generics are mainly sold in developing countries, so it is not a threat to the US’s core markets. There could also be profitable partnership opportunities, if Indian firms would offer their expertise about trading in Africa (whose pharmaceutical market will be worth an estimated US$ 30 billion by 2016) in exchange for R&D resources. (*scidev.net*, 28 Aug 2015)

➢ In January 2015, the Indian government consulted on tighter laws on tobacco control, eg, raising the minimum smoking age to 21 years, and banning the sale of single cigarettes, which account for 70% of sales. Each year, 1 million Indian people die from smoking–related causes–making it one of the most common causes of death. India spends US$ 15.9 billion on treating smoking–related illness each year–almost six times the amount raised through tobacco tax revenues. Taxes on Indian cigarettes have increased sharply to 60% of the retail price–approaching the WHO recommendation of 75%. However, India’s traditional bidi cigarettes have largely escaped these curbs. Bidis are taxed at 7% of their retail price, although bidi cigarettes are riskier than conventional cigarettes as they are inhaled more deeply. According to the Public Health Foundation of India, doubling taxes on bidis could cut consumption by 40%, and increase tax revenue by 22%. In addition, people making bidi cigarettes are generally under–paid, and face a range of health problems (including cancer), as masks and gloves are not used. (*Indiaspend.org*, 1 Sept 2015)

➢ In an interview with India’s *Economic Times*, Bill Gates spoke of how India can make progress in reaching the new Sustainable Development Goals (SDG). He praised India’s progress in achieving some of the MDG targets, and outlines the importance of quality primary health care for India, which must be robust and fully functional. He believes that this will help close the gap on preventable deaths in India, which disproportionately affects the poorest people. Overall, he highlighted the MDGs’ tangible results, such as the sharp falls in child and maternal mortality. He is optimistic that the SDGs will build on this to eradicate extreme poverty and hunger, foster inclusive economic growth and combat climate change by 2030. (*Economic Times of India*, 30 Sept 2015)

## THE AMERICAS

➢ Cuba has become the first country to receive WHO validation for eliminating (ie, a reduction in transmission to a level that does not threaten public health) mother–to–child transmission of HIV and syphilis. PAHO has worked with Cuba and other countries to roll out a programme to eliminate this transmissions. Measures include improved access to prenatal care, more screening for pregnant women and their partners, caesarean deliveries and substituting breastfeeding. Globally, 1.4 million HIV–positive women become pregnant each year, and antiretroviral treatment reduces the risk of transmission from 15–45% to 1%. In welcoming the validation, Michel Sidibé, the UNAIDS executive director said that “this is a celebration for Cuba and a celebration for children and families everywhere. It shows that ending the AIDS epidemic is possible and we expect Cuba to be the first of many countries coming forward to seek validation that they have ended their epidemics among children.” (*IPS*, 30 June 2015)

➢ From 2002 to 2013, US deaths from heroin overdoses rose from 0.7 to 2.7 per 100 000 of population. Rates of use, abuse and dependency increased to 517 000 by 2013–a 150% increase from 2007. Rates are highest amongst males, those aged 18–25 years, people living in urban areas, poorer people and people without health coverage. However, rates increased across all groups over the period. Most overdose deaths involved multiple drugs, and the relationship between heroin and cocaine use is particularly strong. The increased availability, lower price and higher purity of heroin is a potential contributor to the rise, and it is vital that public health works with law enforcement to counter the crisis. Any responses must focus on reducing the rates of non–medical use opioid pain relievers, as the rates of initiating heroin use in this group are 19 times higher than those outside it. It is also vital to improve health insurance coverage to heroin users, in order to reduce usage and addiction, and to reduce HIV and hepatitis transmission. (*CDC*, 7 Jul 2015)

➢ Bioven, a Malaysian biotech company, will seek an Initial Public Offering (IPO) to raise funds for marketing a promising cancer drug which was developed in Cuba. The IPO is expected to raise US$ 30–35 million for the drug, which targets non–small cell lung cancer. Phase III trials involving 419 patients across 10 countries are under way, and a separate trial will take place in the USA under the guidance of the Roswell Park Cancer Institute. More clinical development is under way at the Beatson Institute for Cancer Research in Glasgow, UK. (*Xinhua*, 30 Jul 2015)

Former President of the USA, Jimmy Carter, recently announced that his cancer had spread to his brain. He also spoke about his non–profit Carter Center’s work to combat guinea worm disease. The Carter Center began this work in 1986, when there was 3.5 million cases across 21 countries. Infection from the water–borne guinea worm is painful, and can take months of recovery. Thanks to educational campaigning on the importance of filtering water and avoiding submerging guinea worm lesions in water, there are now only 11 cases left worldwide. When it is wiped out, guinea worm disease will join smallpox as the second human disease to be eradicated. “I’d like the last guinea worm to die before I do,” said Mr Carter. (*Huffington Post*, 21 Aug 2015)

➢ According to PAHO, malaria cases and deaths have fallen sharply across Latin America, with Brazil, Honduras and Paraguay showing the most progress. Cases have fallen by nearly 70%, from 1.2 million in 2000 to 375 000 in 2014, and malaria deaths fell by nearly 80% over the same period. PAHO praised Brazil’s national prevention programme. Worldwide, control and prevention techniques have led to a 60% reduction in mortality rates since 2000. However, 438 000 people died from malaria in 2014–and 91% were in sub–Saharan Africa. The UN aims to cut the numbers of malaria cases and deaths by a further 90% by 2030, which will require an increase of funding from US$ 2.7 billion to US$ 8.7 billion. (*Thomson Reuters Foundation*, 6 Nov 2015)

